# A Web-Based Mindfulness-Based Cognitive Therapy for Couples Dealing With Chronic Cancer-Related Fatigue: Protocol for a Single-Arm Pilot Trial

**DOI:** 10.2196/48329

**Published:** 2023-11-06

**Authors:** Fabiola Müller, Sophie van Dongen, Rosalie van Woezik, Marijke Tibosch, Marrit A Tuinman, Melanie P J Schellekens, Jean-Philippe Laurenceau, Marije van der Lee, Mariët Hagedoorn

**Affiliations:** 1 Department of Health Psychology University Medical Center Groningen University of Groningen Groningen Netherlands; 2 Scientific Research Department Helen Dowling Institute Bilthoven Netherlands; 3 Department of Medical and Clinical Psychology Tilburg University Tilburg Netherlands; 4 Department of Psychological and Brain Sciences University of Delaware Newark, DE United States

**Keywords:** acceptability, cancer, chronic cancer-related fatigue, couple intervention, eMBCT, fatigue, feasibility, partners, pilot trial, web-based mindfulness-based cognitive therapy

## Abstract

**Background:**

Chronic fatigue is a common symptom among patients who have been treated for cancer. Current psychosocial interventions typically target the patient alone, despite growing evidence suggesting that a couples’ approach can increase and broaden the efficacy of an intervention. Therefore, based on an existing web-based mindfulness-based cognitive therapy for patients, the couple intervention COMPANION was developed.

**Objective:**

The primary objectives of this study are to determine the acceptability of COMPANION and its potential efficacy in reducing fatigue in patients with cancer. Our secondary objectives are to examine the feasibility of the trial procedures and the potential working mechanisms of the couple intervention.

**Methods:**

We will conduct a single-arm pilot trial for couples (ie, patients with cancer with chronic fatigue and their partners). All couples are allocated to the web-based couple intervention that consists of psychoeducation, mindfulness, and cognitive-behavioral exercises. The 9 sessions of the intervention are supervised remotely by a trained therapist. Patients and partners will complete questionnaires before starting the intervention (T0), 2 weeks after completing the intervention (T1), and 1 month after T1 (T2). They will also fill out weekly diaries during the intervention period. A subsample of patients (n≈5) and partners (n≈5) as well as all the therapists providing COMPANION will participate in the final focus groups. Benchmark values have been defined to determine the acceptability (ie, ≥60% of couples complete the intervention and/or ≥70% of the participants are satisfied with the intervention) and potential efficacy (ie, a significant improvement in fatigue and/or a clinically relevant improvement in fatigue in 45% of the patients between T0 and T1) of the intervention. The trial procedures are deemed feasible if an average of at least three couples are included per recruiting month and/or adherence to the assessments is at least 65% for T1 and the diaries and 60% for T2. To establish potential working mechanisms, changes in affect, sleep, catastrophizing, partner communication and interactions, self-efficacy, mindfulness, and closeness will be examined. Quantitative outcomes will be interpreted along with the results from the focus groups.

**Results:**

Data collection is expected to be completed by March 2024.

**Conclusions:**

This pilot trial will test the first web-based mindfulness-based cognitive therapy for couples targeting chronic cancer-related fatigue. Findings will indicate whether proceeding with a randomized controlled trial is warranted.

**Trial Registration:**

ClinicalTrials.gov NCT05636696; https://clinicaltrials.gov/study/NCT05636696

**International Registered Report Identifier (IRRID):**

DERR1-10.2196/48329

## Introduction

### Background

Chronic cancer-related fatigue (CCRF) is a common symptom among patients treated with curative [1-4] as well as palliative intent [5-7]. It can persist for many years and has a profound negative impact on patients’ quality of life [2,8,9]. Its etiology is likely multifactorial and includes several biological pathways [10,11]. According to the cognitive-behavioral model of CCRF, cancer and its treatment initially trigger fatigue, while cognitive-behavioral variables (eg, negative cognitions about fatigue and disrupted activity patterns) explain its persistence after completion of treatment [12-14]. Based on this model, cognitive-behavioral and mindfulness-based therapies have been developed to address CCRF. While these and other interventions have been shown to effectively alleviate patients’ fatigue [15-19], they are directed at the patient with cancer alone, despite growing evidence for the importance of involving patients’ partners in the treatment of CCRF (ie, a couples’ approach to treatment).

CCRF is experienced in the context of patients’ close relationships in multiple ways. First, the way patients and their partners interact in daily life is related to patients’ fatigue outcomes. For example, a daily diary study among survivors of cancer and their partners showed that partners’ facilitative reactions (eg, encouragement to be active) were related to better fatigue outcomes, while solicitous reactions (eg, taking over the patient’s chores) as well as ruminative conversations—fueled by patients and their partners maladaptive cognitions—were related to worse outcomes during the day [20,21]. This suggests that interventions encouraging adaptive daily interactions between couple members have the potential to contribute to better fatigue outcomes for the patient. Second, the degree to which patients benefit from cognitive behavioral therapy for fatigue appears to be related to partner and relationship variables. That is, a recent study [22] among patients with chronic fatigue syndrome showed that relationship dissatisfaction among patients and high fatigue in their partners are associated with less improvement in fatigue severity after therapy. Third, ample research shows that the cancer experience also impacts intimate partners. In particular, partners have been shown to experience substantial distress [23,24], which is positively related to patient fatigue [25,26], suggesting that an effective fatigue intervention can also benefit partners. Fourth, the aforementioned diary study [20] suggests that the way a partner deals with fatigue in the couple’s daily life is not only related to fatigue outcomes but also patients’ relationship satisfaction, with solicitous and facilitative responses being related to higher relationship satisfaction. Jointly, the evidence suggests that targeting the couple instead of the patient alone has the potential to increase the intervention’s effect on patient fatigue and broaden its impact to alleviate partner distress and benefit the couple’s relationship.

Based on this and studies indicating beneficial effects of interventions for couples coping with cancer in general [27-30], the study team has developed a couple intervention for dealing with CCRF called COMPANION (Dutch: Samen Minder Moe). The intervention is based on a web-based mindfulness-based cognitive therapy (eMBCT) that has been shown to be effective in reducing patient fatigue [31].

### Research Aims

Given the relative novelty of this interventional approach, conducting a pilot trial is warranted. The primary aims of this pilot trial are (1) to determine the acceptability of the couple intervention and (2) to investigate its potential efficacy in reducing patient fatigue. Secondary aims are (3) to examine the feasibility of the trial procedures and (4) to determine the potential working mechanisms of the couple intervention.

## Methods

### Design

**Figure 1:**
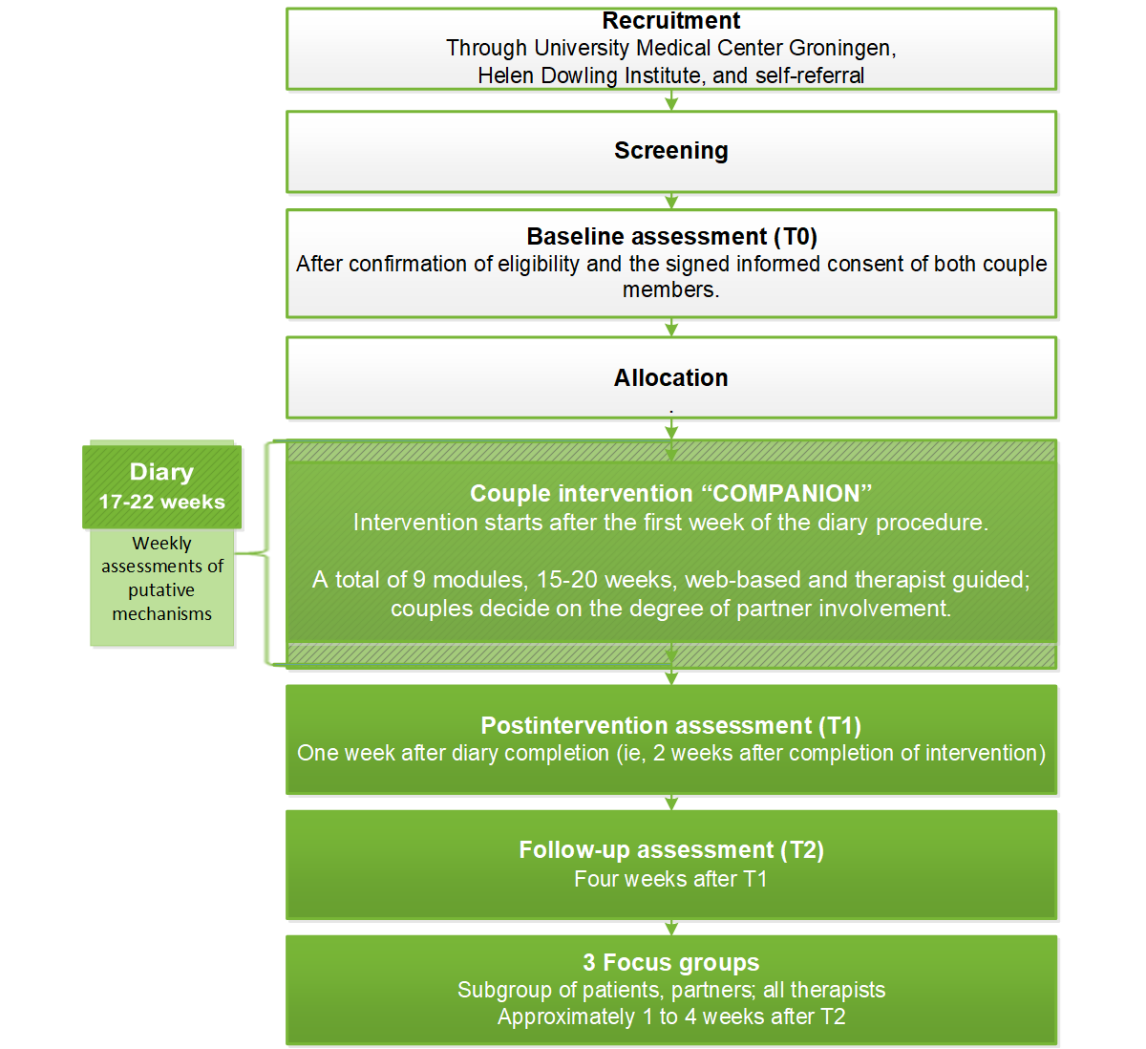
The study flowchart.

This study is a single-arm pilot trial among patients with cancer with CCRF and their partners. All included couples will be allocated to receive the COMPANION intervention, with a planned duration of 15-20 weeks. Couples will complete questionnaires and weekly diaries. Trial participation is estimated to take 6-8 months in total. Figure 1 presents a flowchart of the study.

### Participants

Eligible participants are couples in which one member was diagnosed with cancer, completed cancer treatment for at least 3 months (excepting hormone therapy), and has been experiencing severe fatigue for at least 3 months. See Textbox 1 for an overview of all inclusion and exclusion criteria.

**Inclusion and exclusion criteria for the study.**

**Inclusion criteria**

- The patient has received a cancer diagnosis (all malignancies are included).
- The patient completed cancer treatment with either curative or palliative intent ≥3 months earlier. Patients who currently receive hormone therapy are eligible.
- The patient experiences severe levels of fatigue (a score of ≥35 on the Checklist Individual Strength, subscale fatigue severity) at the screening.
- The patient has been experiencing severe fatigue for ≥3 months (as self-reported by the patient).
- The patient was 18 years of age or older at disease onset.
- The partner is 18 years of age or older.
- Both couple members live together.
- Both couple members have good command of the Dutch language (checked implicitly during registration).
- Both couple members have adequate computer literacy and have access to an internet-connected computer, laptop, or tablet (based on self-report).
- Both couple members agree to participate in the research.

**Exclusion criteria**

- The patient is currently following an evidence-based therapy for chronic cancer-related fatigue (ie, cognitive behavioral therapy, mindfulness-based therapy, exercising, or physiotherapy) as self-reported at the telephone screening.
- The patient has a condition that can explain his or her fatigue and is potentially treatable (eg, anemia).
- The therapist decides, based on information collected during the intake session, that the intervention is not suitable for the couple. Criteria that will be considered include, but are not limited to:
  The presence of severe psychiatric morbidity such as suicidal ideation or psychosis (as assessed by the therapist at the intake session). Mild depression is not an exclusion criterion. A score of ≥20 on the hospital anxiety and depression scale at T0 is considered indicative of depression [32]. Therefore, if the patient or the partner scores ≥20, the therapist will determine at intake whether the participant has suicidal ideation or has another severe psychiatric morbidity. A participant (and thus the couple) will be excluded if, according to the therapist, that is the case.
  The presence of substance abuse, except for smoking.

### Recruitment

Recruitment will take place at the University Medical Center Groningen (UMCG), the Helen Dowling Institute (HDI), and through self-referral. At the UMCG, patients will be invited to the study by their oncologist or nurse, either at a follow-up appointment or during a phone call. Couples who indicate interest will be contacted by the research assistant. At the HDI, the research assistant will contact patients who are on the waiting list to receive patient-centered eMBCT and have given permission to be contacted for research purposes. Recruitment through self-referral will be encouraged by social media posts, newspaper articles, the panel of Kanker.nl [33], and flyers to be distributed at psychosocial care centers for people affected by cancer (Dutch: IPSO centra). Interested couples will be directed to the project website [34], where they can register.

### Screening

The research assistant will schedule a web-based call with all interested couples to further inform them about the study and screen their eligibility. During the screening process, reasons for ineligibility and decline of participation will be recorded in the recruitment log as they inform our secondary aim (ie, feasibility of trial procedures). Upon obtaining informed consent from the patient with cancer and their partner, the patient will receive a link through email to a survey to confirm fatigue- and cancer-related eligibility criteria.

### Assessments

Participants will complete questionnaires before starting the intervention (T0), 2 weeks after completing the intervention (T1), and 1 month after T1 (T2). Patients and their partners will also complete weekly diaries, starting 1 week before the intervention and ending 1 week after the intervention is completed. All questionnaires and diaries will be provided on the web through a secure, web-based application called REDCap (Research Electronic Data Capture) [35]. Patients and their partners will receive a link sent to their personal email and will be encouraged to respond independently from each other. In the event of nonresponse to the questionnaire, participants will receive a reminder email after 7 days and a second reminder after another 7 days of nonresponse. In case a diary assessment is missed, participants will receive a reminder the next day, and if 2 consecutive diaries have not been responded to, the assistant will call the participant to discuss possible problems. Therapists will also keep a therapist log to register data on intervention delivery, and the research assistant will keep a recruitment log to register data on recruitment and study adherence. A subsample of 5 patients with cancer and 5 partners (anticipated sample sizes), as well as all therapists providing the COMPANION intervention, will be invited to participate in separate focus groups. Patients and partners will be sampled purposefully (ie, based on high vs low levels of adherence or intervention satisfaction) and can be invited independently from each other.

### Sample Size Calculation

The study is powered to test the potential efficacy of the couple intervention in reducing patient fatigue (ie, the second primary research aim). Patients’ baseline (T0) and postintervention (T1) scores on the Checklist Individual Strength, subscale Fatigue Severity (CIS-fatigue) will be analyzed with a 1-tailed matched pairs *t* test. G*Power analysis indicated that 27 patients are required to detect a medium effect (0.5) with an α of .05 and a power of 0.8 (1-tailed). We expect CIS-fatigue scores at T1 to be significantly lower than CIS-fatigue scores at T0. We will conduct a 1-sided test given the gain in power as compared to a 2-sided test and evidence that an increase in mean fatigue is unlikely to occur [31]. Based on the drop-out rate in our previous trial [31] and a recent review of the attrition rate in couple-based interventions for cancer [36], we assume a 20% drop-out rate. Hence, we will aim to include a total of 34 couples.

### Intervention

COMPANION is a couple intervention developed from an evidence-based patient-centered eMBCT that aims to change the patient’s behavioral and cognitive reactions to cancer-related stressors, including fatigue [31]. Like the patient-centered eMBCT, COMPANION is web-based and guided remotely by a trained therapist. However, COMPANION also involves the patient’s partner in the treatment. The intervention consists of 9 sessions, which take approximately 15 to 20 weeks to complete (ie, the intervals between sessions are flexible to accommodate holidays and other commitments). Patients will have access to a secure web-based platform where they will receive written information about a specific theme for each session. Partners will have access to these materials through the patient. Table 1 provides an overview of all 9 sessions. Participants will be provided with audio files to practice different mindfulness-based cognitive-behavioral exercises each session. Patients will document their experiences in their personal log and perform exercises for approximately 30 minutes, 6 days a week. The exercises comprise topics such as relaxation, eating with awareness, moving with awareness, 3-minute breathing exercises, or meditation. Apart from the video call consultations, communication with the therapist will be mostly asynchronous, meaning that the therapist reads the experiences in the logs and provides feedback once a week. Adaptations compared to the patient-centered version were based on the findings from the preceding needs assessment among patients, partners, and therapists (van Dongen et al, unpublished data, 2023). These adaptations mainly concerned: 3 video call consultations between patient, partner, and therapist; psychoeducation for the partner; additional exercises for patient and partner (to be performed together or independently); and an extra session focused on the couple’s mutual relationship and mindful communication about fatigue. Together with their therapist, couples can decide how and to what extent their partner will be involved.

**Table 1:** Modules of COMPANION.

Theme of module	Web-based psychoeducation	Exercises
Module 1: Fatigue and the automatic pilot	Automatic pilot Coping with fatigue Patients formulate treatment goals	Practice relaxation and awareness of the body with an audio file “bodyscan” Register experiences with the “bodyscan” Register experiences with eating with awareness
Module 2: Practice being open-minded	Coping with pain and fatigue Handling thoughts during breathing exercise Tips for better sleep quality	“Bodyscan” with muscle tension Breathing exercise Register experiences with these exercises Register awareness during a specific activity Register awareness during pleasant moments
Module 3: Dealing with boundaries	Awareness of handling physical and emotional boundaries Cultivating acceptance	Moving with awareness and a 3-minute breathing exercise, alternated with previous exercises Register experiences with these exercises Register awareness about what happens when meeting boundaries
Module 4: Dealing with stress	Patience Recognizing automatic negative cognitions Recognizing daily stress-inducing experiences and their emotional impact	Using the senses (hearing, seeing, and feeling) with awareness Alternating with previous exercises
Module 5: Communication	How to communicate about cancer-related fatigue How to communicate mindful of your own and others’ thoughts and feelings	Mindful communication exercise Alternating with previous exercises
Module 6: Dealing with feelings	Accept things as they are Coping with negative emotions through acceptance	Allowing intense feelings by focusing on present awareness of sensations in the body Alternating with previous exercises
Module 7: Dealing with thoughts and anxiety	Dealing with thoughts and fears Interaction between thoughts, emotions, and behaviors Physiology of fear	Becoming aware of automatic negative thinking Making a list of these thoughts Alternating with previous exercises
Module 8: Self-care	Self-care	Compose own practice schedule by choosing from previous exercises Making a list of helpful cognitions
Module 9: From stress and fatigue to strength	Patients continue practice with their own practice schedule	Writing about what helps their practice Formulation of potential pitfalls in the future and composition of self-management strategies

### Therapist Training and Treatment Integrity

Therapists at the HDI will deliver COMPANION. They all fulfill the criteria established by the United Kingdom Mindfulness-Based Teacher Therapist Network Good Practice Guidelines for teaching mindfulness-based interventions [37]. They will be trained in providing the couple intervention by an experienced therapist and a researcher from the COMPANION project team. This training starts with a preparatory self-study assignment using the web-based COMPANION treatment environment (a web portal for therapists). Next, therapists participate in an interactive training session addressing the content of the new therapy, the role of the therapist within this new therapy (including exercises and suggestions for involving the partner and giving therapeutic feedback [38]), and the design and procedures of the COMPANION pilot trial. All information is documented in a detailed written manual for therapists. Regular supervision will take place during the pilot trial, led by a member of the COMPANION project team who is a registered supervisor at the Dutch Society for Cognitive Behavioral Therapy. This supervisor has 15 years of experience in psycho-oncology, including 12 years of work experience with the patient-centered eMBCT for CCRF.

### Measures

Table 2 provides an overview of all the constructs to be assessed. For conciseness, we have only described the key measures. Where possible, we chose validated and widely used instruments in the field. Table 2 provides references for measures not described here.

**Table 2:** Overview of instruments, questionnaires, and assessment time points. Unless otherwise indicated, both patients and their partners complete questionnaires, and they report on their own health, perception, and state. Partner version of scales are own adaptations.

Construct		Instrument or type of assessment	Assessment time points				
			Screening^a^	T0^b^	T1^c^	T2^d^	FG^e^
**Personal characteristics**							
	Demographics	Standard items	✓	✓			
	Cancer-related characteristics^f^	Standard items	✓	✓			
	Fatigue onset^f^	Developed item	✓				
	Fatigue duration^f^	Developed item	✓				
	Current CCRF^g^ care use^f^	Developed item	✓				
**Primary and secondary outcomes**							
	Intervention completion	Developed items, self-report and therapist log			✓		
	Intervention satisfaction	Developed items based on [39,40]			✓		
	Intervention experiences	Not applicable					✓
	Partner involvement	Developed item, self-report and therapist log			✓		
	Fatigue severity^h^	CIS-fatigue^i^	✓	✓	✓	✓	
**Potential** **mediators: mindfulness- and cognitive-behavioral variables**							
	Self-efficacy	SES^j^ or SES partner version		✓	✓		
	Catastrophizing	J-FCS^k^ or J-FCS partner version		✓	✓		
	Mindfulness	FMI^l^		✓	✓		
**Other relevant measures**							
	Emotional well-being	HADS^m^		✓	✓	✓	
	Relationship satisfaction	MMQ-marital satisfaction^n^		✓	✓	✓	
	Caregiver burden regarding patient fatigue^o^	ICQ^p^, partner version		✓	✓		
	Perceived change in fatigue^f^	Developed item based on [41]			✓		
	Adverse events	Developed items based on [41], self-report and therapist or recruitment log			✓		
	Supportive care use for mental health issues	Developed items based on [41]		✓			

^a^Some screening items will be assessed verbally on the phone (eg, relationship status), others through a questionnaire (eg, fatigue items).

^b^T0 is baseline.

^c^T1 is 2 weeks after completion of the intervention.

^d^T2 is 1 month after T1.

^e^FG: focus group.

^f^Patient only.

^g^CCRF: chronic cancer-related fatigue.

^h^For partners at baseline and T1 only.

^i^CIS-fatigue: Checklist Individual Strength, subscale Fatigue Severity [42,43].

^j^SES: Self-Efficacy Scale [44].

^k^J-FCS: Jacobsen Fatigue Catastrophizing Scale [45].

^l^FMI: Freiburg Mindfulness Inventory Short Form [46].

^m^HADS: Hospital Anxiety and Depression Scale [47,48].

^n^MMQ: Maudsley Marital Questionnaire, subscale Marital Satisfaction [49,50].

^o^Partner only.

^p^ICQ: Illness Cognition Questionnaire [51].

Demographic characteristics will be assessed at the screening and T0 and include self-reported age, sex, level of education, occupational status, relationship status and duration, cohabitation status and duration, and comorbidities.

Patients’ cancer-related variables are self-reported and include cancer type, prognosis, presence of metastasis, treatment received, time since diagnosis, and time since treatment completion.

Intervention adherence will be assessed at T1, as self-reported by the patients and as registered by the therapists in their therapy log. The item used reads: “Did you/your client complete all sessions of the COMPANION therapy?” Answer categories are (1) “Yes, I/my client completed all sessions of the intervention” and (2) “No, I/my client stopped after session ____ (fill in the blank).” Reasons for stopping can be recorded in a free text field. By design, partners’ involvement in the intervention is flexible, as this was an important requirement derived from the preceding needs assessment study (van Dongen et al, unpublished data, 2023). We consider receiving 6 sessions to be the minimal therapeutic dose. Therefore, intervention completion is achieved when a patient receives ≥6 out of 9 sessions, as reported by the patient at T1 and/or in the postintervention therapist log, and the partner did not drop out from the intervention.

Intervention satisfaction will be assessed with items based on those of other pilot trials for patients and their loved ones [39,40]. For both patients and partners, a total of 2 items will be used to assess satisfaction with the intervention overall (ie, “Overall, how satisfied are you with the COMPANION therapy?”) and satisfaction with the couple approach (ie, “How satisfied are you with the possibility of jointly participating in the COMPANION therapy?”). Additional items assess satisfaction with specific aspects of the intervention. Items are scored on a 7-point Likert scale; scores 5 to 7 represent (high) satisfaction.

Fatigue severity will be assessed using the CIS-fatigue. A total of 8 items assess fatigue severity during the past 2 weeks (eg, “I feel tired”) and are scored on a 7-point Likert scale, ranging from (score 1) “Yes, that is true” to (score 7) “No, that is not true.” A higher sum score indicates more severe fatigue (range 8-56), with a score of ≥35 indicating severe fatigue in patients with cancer [42,43]. A clinically relevant change is operationalized as a difference of 6 points [52].

Recruitment rates: the research assistant will complete a recruitment log. The following information will be collected: (1) number of patients approached for recruitment (through the UMCG and HDI); (2) number of participants (ie, patient and/or their partner) interested in participation; (3) number of included couples; and (4) number of couples dropping out from the intervention and study. Along with these rates, reasons will be recorded. The recruitment rate is defined as the average number of couples included per month.

Adherence to the study protocol will be recorded by the research assistant in the recruitment log in terms of the number and percentage of questionnaires completed. Completion is defined as having responded at least to the items assessing the outcome measures.

Potential mechanism variables will be assessed in the weekly diary and include affect, sleep, catastrophizing, partner communication, partner interactions, self-efficacy, mindfulness, and closeness. Items are based on and adapted from a daily diary study among survivors of cancer and their partners [20,21,53] and existing scales. Table 3 provides an overview of the items.

**Table 3:** Concepts assessed with the diary method. Participants complete weekly diaries starting the week before the intervention, during the course of the intervention, and the week following the end of the intervention (ie, a total of 17-22 weeks) to assess potential working mechanisms of COMPANION.

Constructs		Items and instruments	Time frame
**Outcome variables**			
	Fatigue severity^a^	1 item, as in [20,21,53]	Momentary^b^
	Fatigue severity^c^	4 items based on SFQ^d^	Last week^e^
	Intervention adherence	1 developed item	Last week
	Partner involvement	2 developed items	Last week
**Potential mechanism variables**			
	Affect	5 items, shortened from [53]	Momentary
	Sleep	2 items, as in [53]	Last night^f^
	Catastrophizing	4 items, as in [21,53]	Last week
	Partner communication	3 items, based on [21]	Last week
	Partner interactions	6 items, shortened from [20]	Last week
	Self-efficacy	3 items, based on the SES^g^	Last week
	Mindfulness	4 items, based on the FMI^h^	Last week
	Closeness	2 developed items	Last week

^a^Patient only.

^b^Items ask the participants to report their current state (eg, “How fatigued do you feel right now?”).

^c^Partners report on their perception of patients’ fatigue.

^d^SFQ: Short Fatigue Questionnaire [54].

^e^Items ask the participants to report their state from the previous week.

^f^Items ask the participants to report their state from the previous night.

^g^SES: Self-Efficacy Scale [44].

^h^FMI: Freiburg Mindfulness Inventory Short Form [46].

Weekly fatigue severity in patients will be assessed with the 4-item Short Fatigue Questionnaire (SFQ) [54], a shortened version of the CIS-fatigue. The time frame has been adapted from the original past 2 weeks to the previous week, and items are formulated in the past tense (eg, “I felt tired”).

Current fatigue severity will be assessed in the weekly diaries with 1 item, “How fatigued do you feel right now?” that is scored on an 11-point Likert scale ranging from (score 0) “not at all” to (score 10) “as fatigued as I could be” [20,21,53].

### Analyses

Analyses will be conducted in SPSS (IBM Corp) and Mplus (Muthén & Muthén).

#### Descriptive Statistics

Descriptive statistics will be presented in a table. Data for patients with cancer and their partners will be presented separately (with the exception of couple characteristics such as relationship status). A study flowchart will show the number of couples screened, eligible, and included, as well as reasons for noneligibility and dropout.

#### Benchmark and Critical Values

This pilot trial is designed to assess the acceptability, potential efficacy, and potential working mechanisms of COMPANION and to determine the feasibility of trial procedures. To establish whether a subsequent randomized controlled trial (RCT) is justified, we have set benchmarks and critical values. Benchmark values represent the lower values of what we deem desirable to achieve. Critical values are defined as half of the benchmark values and represent potential problems with the outcome assessed. These benchmarks and critical values are based on comparable literature and our clinical and research experience. As each trial is unique (eg, in terms of study design, target population, intervention content, and duration), benchmark values are set rather conservatively (ie, similar to or lower than in comparable studies). The benchmark and critical values will not serve as definitive thresholds to determine whether a larger RCT is justified but will be interpreted along with the other outcomes, focus group data, and the recruitment log. Table 4 provides an overview of the key outcomes, their operationalizations, benchmark, and critical values.

**Table 4:** Overview of outcome measures, their operationalizations, benchmark, and critical values.

Outcome		Operationalization	Data	Benchmark values	Critical values
**Acceptability of COMPANION**					
	Intervention adherence	Percentage of couples in which the patient completed the intervention, that is, followed at least 6 out of 9 sessions, and the partner did not drop out	T1 (patients) and/or therapist log	≥60% of couples completed the intervention	<30% of couples completed the intervention
	Intervention satisfaction	Percentage of patients and partners responding to T1 who are satisfied with the couple approach and the intervention overall (score ≥5 on a 7-point Likert scale)	T1 (patients and partners)	≥70% of patients and ≥70% of partners are satisfied with the intervention overall and/or the couple approach	<35% of patients and <35% of partners are satisfied with the intervention overall and/or the couple approach
**Potential efficacy for patient fatigue**					
	Significant change in patient fatigue	Statistically significant decrease from CIS-fatigue_T0_^a^ to CIS-fatigue_T1_ (intention-to-treat).	T0 and T1 (patients)	Significant improvement in patient fatigue (*P<*.05, intention-to-treat)	N/A^b^
	Clinically relevant improvement	Percentage of patients in which CIS-fatigue_T0_–CIS-fatigue_T1_≥6.	T0 and T1 (patients)	≥45% of patients improved (intention-to-treat)	<23% of patients improved (intention-to-treat)
**Feasibility of trial procedures**					
	Rates related to the recruitment procedure	Recruitment rate: average per month, averaged across recruitment strategies.	Recruitment log	≥3 couples included per month	<1.5 couples included per month
	Adherence to the T1 and T2 questionnaires	Percentage of patients and partners completing and returning T1 and T2.	T1 and T2 (patients and partners), recruitment log	≥65% of patients and partners completed T1 and ≥60% of patients and partners completed T2	<33% of patients and partners completed T1 and <30% of patients and partners completed T2
	Adherence to the diary protocol	Percentage of diaries completed by patients and partners.	Diary (patients and partners), recruitment log	≥65% of diaries completed by patients and partners	<33% of diaries completed by patients and partners
**Potential working mechanisms**					
	Changes in fatigue and potential mechanisms over time	Co-occurrence of time slopes of potential mechanisms with time slope of fatigue.	Diary (patients)	Targeted cognitions and behaviors improve (ie, time slopes are significantly different from 0); improvements co-occur with improved fatigue (standardized covariance is significant, *P*<.05)	N/A

^a^CIS-fatigue: Checklist Individual Strength, subscale Fatigue Severity.

^b^Not applicable.

#### Primary Aims

Acceptability of the couple intervention will be determined in terms of adherence to and satisfaction with the couple intervention. The benchmark value for adherence is based upon the rates of the trial that tested the patient-centered intervention (ie, 38% of intervention dropout [31]) and on comparable web-based couple interventions [55,56]. Couples will not be considered in cases where the investigator decides to withdraw the couple or a change in health status occurs that interferes with participation. The benchmark value for satisfaction is based upon the couple intervention trial by McDonnell et al [40], in which acceptability with different intervention components ranged between 75% and 100% for patients and family members.

The potential efficacy of the couple intervention will be determined in terms of a statistically significant decrease and a relevant improvement (≥6-point decrease on CIS-fatigue) in patient fatigue between T0 and T1. Both intention-to-treat analyses (ie, including all couples allocated) as well as per-protocol analyses (ie, including only couples who completed COMPANION as defined above) will be performed. The benchmark value for the percentage of clinically relevant improvement is based on our previous trial, in which 49% of the patients allocated to patient-centered eMBCT benefitted in terms of reduced fatigue (intention-to-treat; using a stricter criterion to define improvement as used here) [31].

Missing values will be replaced with multiple imputations using chained equations. The imputation model will include demographic and clinical variables as assessed at baseline. In sensitivity analyses, completer analyses will be performed (ie, including only cases with nonmissing CIS-fatigue_T0_ and CIS-fatigue_T1_ values). Of note, the study evaluating the patient-centered intervention included only curatively treated patients. It might be that the formulated benchmark values for efficacy are too strict for patients with cancer recurrence or reinitiation of treatment. If applicable, we will therefore calculate the efficacy outcomes with and without these patients included.

#### Secondary Aims

The feasibility of the trial procedures will be determined in terms of the recruitment rate. In our trial testing patient-centered eMBCT, on average, a total of 6 patients were included per month [31]. Given the known challenges in recruiting dyads for interventional research [36,57-59], the benchmark value is set lower, even lower than the number required (ie, 3,8) to reach our target sample size within the planned 9-month recruitment period.

The feasibility of the trial procedures will also be assessed in terms of adherence to the study protocol. In our previous trial, 73% of patients completed T1 and T2 [31]. In this trial, the follow-up period is shorter than in comparable trials (ie, 4 weeks). Still, given the intensity of the diary assessments (ie, up to 22 diaries), we set our benchmark value for completion of both assessments conservatively. In our observational diary study among fatigued survivors of cancer and their partners, compliance with the evening diary exceeded 90% [21]. While assessments in this study were also performed in the morning and noon, the diary period lasted only 2 weeks. It seems likely that the completion rate in this study is lower as the diary procedure is embedded in intensive treatment and the diary period covers several months; a study that had a diary duration of 6 months had a completion rate of 69% [60].

Based on the cognitive-behavioral model of fatigue and previous research [20,21,53,61], the potential mechanism variables are affect, sleep, catastrophizing, partner communication, partner interactions, self-efficacy, mindfulness, and closeness. A variable is established as a potential working mechanism of the couple intervention if its change (ie, improvement) over the course of the diary period covaries with decreases in weekly fatigue. Multilevel growth curve analysis will be applied to model the time slopes of the potential mechanisms and weekly fatigue over the entire diary period. We will explore both linear and nonlinear time trends and consider modeling random intercepts and random slopes. An improvement in fatigue and maladaptive constructs such as catastrophizing is indicated by a decrease over time. An improvement in adaptive constructs such as self-efficacy and mindfulness is indicated by an increase over time. For mechanism variables pertaining to partner communication and partner interactions, an improvement might be indicated by a decrease or increase. For some couples, talking (temporarily) more about fatigue is adaptive, while for others, talking (temporarily) less is adaptive. Covariances between the time slopes of the presumed mechanisms and the time slope of daily fatigue will be estimated to identify whether the temporal changes co-occur. A variable is established as a potential working mechanism in case the standardized covariance between the slope change factor for the outcome and that of the presumed mediators is significant (using a *z* test where ±1.96 is significant at the .05 level). Models will be run for weekly fatigue as well as weekly-reported momentary fatigue.

#### Explorative Analyses

First, we will explore whether there are differences between couples with low versus high partner involvement. A comparison will be performed on personal and couple characteristics. Second, we will explore whether the degree of partner involvement is related to the potential effect on patient fatigue, intervention adherence, and satisfaction. Please note that in cases of little variation in partner involvement, we might not be able to conduct these analyses. Third, we will explore the potential efficacy for fatigue severity as assessed at T2. For these analyses, the scores from the T0 and T2 assessments will be used for the statistical test (ie, CIS-fatigue_T0_ and CIS-fatigue_T2_). Fourth, we will explore whether the couple intervention had a positive effect on cancer patients’ and partners’ well-being (as assessed with the Hospital Anxiety and Depression Scale [HADS]) and on couples’ relationship outcomes (as assessed with the subscale marital satisfaction of the Maudsley Marital Questionnaire [MMQ]) at T1 and T2. Lastly, we will explore whether the diary data of partners also show the expected improvement in targeted variables and partner outcomes over time.

#### Analysis of Focus Group Data

Focus groups are held to assess participants’ experiences with following the intervention (patients and their partners) and delivering the intervention (therapists). Barriers and facilitators, as well as ideas for improvement of the intervention and trial procedures, are discussed. The data will be analyzed following the principles of thematic analysis, using open, axial, and selective coding [62]. A total of 2 researchers will independently familiarize themselves with the transcripts of the focus groups and identify initial codes (open coding). These initial codes will be collated into potential themes, described in relation to the coded extracts, and organized into a preliminary coding scheme (axial coding). Emergent themes will subsequently be reviewed and organized according to the main themes, resulting in a final coding scheme (selective coding). Using the constant comparative method [63], we will compare intervention experiences and ideas for improvement both between and within groups (ie, patients, partners, and therapists). Codes, themes, and their interpretations will be regularly discussed within the project team. The qualitative data will be interpreted along with the quantitative data.

### Monitoring

This study is subject to on-site monitoring based on the risk classification “negligible.”

### Ethical Considerations

This study has been approved by the medical ethics committee of the University Medical Center Groningen (registration number 2022/203, NL80201.042.22). The study is registered on ClinicalTrials.gov (NCT05636696). Written informed consent will be obtained from all participating patients with cancer and partners. A separate consent form will be signed by those who participate in the focus groups.

## Results

Data collection is expected to be completed by March 2024.

## Discussion

This pilot trial is designed to assess the acceptability, potential efficacy, and potential working mechanisms of COMPANION, a web-based couple intervention targeting CCRF. The feasibility of the trial procedures will also be determined. Together with qualitative data, a priori benchmarks and critical values for the key outcomes will inform whether conducting a future RCT to test the efficacy of COMPANION as compared to a control condition is warranted. If all or most benchmark values are achieved and qualitative data are reflective of this, progression to a larger RCT without adjustment to the intervention and/or trial is indicated. In the case of less positive results, progression with some adjustments to the intervention and/or trial procedures might be indicated. If few or no benchmark values are reached and qualitative data are reflective of this, it may be decided not to progress to an RCT.

Interventions directed at patients with cancer and their close relatives are becoming increasingly common. However, of those relevant to the proposed pilot study, most either apply a couple’s mindfulness-based approach [64,65] or target cancer-related fatigue [66]. To the best of the authors’ knowledge, there is not yet a mindfulness-based intervention for couples that primarily targets fatigue. There is 1 mindfulness-based (ie, yoga) intervention for patients and their family members that focuses on symptom reduction, with 1 outcome being fatigue reduction [40]; yet, due to the pilot design and small sample size, no conclusions about change in fatigue can be drawn. This lack of studies examining mindfulness-based interventions for couples that primarily target fatigue is surprising given the large evidence base supporting the efficacy of patient-centered mindfulness-based interventions for reducing cancer-related fatigue [16-19,67]. Therefore, the main strength of the proposed pilot trial is that it is the first to test a couple mindfulness intervention primarily targeting CCRF. This couple intervention is based on a patient-centered mindfulness-based intervention that also primarily targets CCRF and has been shown to be effective in reducing it. Further, the adaptation of the patient-centered intervention to the couple was performed with input from relevant stakeholders. Therefore, we expect that the intervention will likely meet the needs of patients and partners. Moreover, the intervention is web-based. Patients, and to a varying degree, their partners, walk through the sessions themselves while being guided remotely by their therapist. This delivery format facilitates the scalability of the intervention due to reduced participant burden and therapist time.

Another strength pertains to the collection of diary data. By measuring potential mechanism variables throughout the treatment, we make the first step toward understanding through which processes the expected beneficial effect may be reached. With this knowledge, the intervention could be made more efficient in the future. Another strength pertains to the collection of qualitative data. These data will be helpful in interpreting quantitative findings and, if needed, providing insight into how the intervention and trial procedures could be improved for a subsequent RCT. Furthermore, applying different recruitment strategies allows us to estimate the most effective ways of including couples. Lastly, we also include patients treated with palliative intent (provided they completed treatment ≥3 months ago; reinitiation of treatment after inclusion is not an exclusion criterion) and we do not exclude participants with comorbidities (barring cases as outlined in Textbox 1), increasing the external validity of the trial.

Several limitations of the pilot trial need to be mentioned. It is a single-arm, uncontrolled trial. Accordingly, results regarding the potential efficacy of COMPANION on patient fatigue and exploratory outcomes will need to be interpreted with caution. Furthermore, due to time constraints, the T2 follow-up assessment is planned only 4 weeks after T1. In a subsequent RCT, a longer follow-up period is needed to assess whether the expected treatment effect will be sustained over time. Adherence to the T2 assessment in this pilot study will therefore likely overestimate adherence to the long-term follow-up in a subsequent RCT.

The authors hope that COMPANION has the potential to positively contribute to CCRF care and benefit patients as well as their partners.

## Abbreviations

CCRF: chronic cancer-related fatigue
CIS-fatigue: Checklist Individual Strength, subscale Fatigue Severity
eMBCT: web-based mindfulness-based cognitive therapy
HADS: Hospital Anxiety and Depression Scale
HDI: Helen Dowling Institute
MMQ: Maudsley Marital Questionnaire
RCT: randomized controlled trial
REDCap: Research Electronic Data Capture
SFQ: Short Fatigue Questionnaire
UMCG: University Medical Center Groningen

## Data Availability

The data sets generated and analyzed during this study are available from the corresponding author on reasonable request.
